# 1,6-Di­bromo­naphthalen-2-ol methanol monosolvate

**DOI:** 10.1107/S1600536813016371

**Published:** 2013-06-26

**Authors:** Marisa B. Sanders, Tania Furkan, Emily Leonard, Benny C. Chan

**Affiliations:** a Department of Chemistry, The College of New Jersey, 2000 Pennington Rd, Ewing, NJ 08628, USA

## Abstract

The naphthol-containing mol­ecule of the title compound, C_10_H_6_Br_2_O·CH_3_OH, crystallized as a methanol monosolvate and is planar to within 0.069 (1) Å for all non-H atoms. In the crystal, mol­ecules are linked by two pairs of O—H⋯O hydrogen bonds, involving the methanol mol­ecule, forming dimer-like arrangements. The crystal structure is further stabilized by π–π stacking [centroid–centroid distance = 3.676 (2) Å] and Br⋯Br inter­actions [3.480 (4) and 3.786 (1) Å], forming a three-dimensional structure.

## Related literature

For information on applications of 1,6-di­bromo-2-napthol, see: Costa *et al.* (2012 ▶); Takeuchi *et al.* (2000 ▶); Kalra & Kumar (2005 ▶). For related structures, see: Rozycka-Sokolowska & Marciniak (2009 ▶). For halogen–halogen inter­actions, see: Zordan & Brammer (2006 ▶); Schlueter *et al.* (2012 ▶); Desiraju & Parthasarathy (1989 ▶).
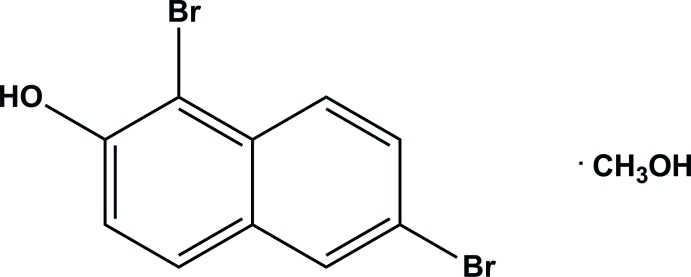


## Experimental

### 

#### Crystal data

C_10_H_6_Br_2_O·CH_4_O
*M* *_r_* = 334.01
Monoclinic, 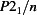

*a* = 3.9971 (4) Å
*b* = 12.4705 (12) Å
*c* = 22.462 (2) Å
β = 92.442 (1)°
*V* = 1118.62 (19) Å^3^
*Z* = 4
Mo *K*α radiation
μ = 7.22 mm^−1^
*T* = 100 K
0.26 × 0.11 × 0.01 mm

#### Data collection

Bruker APEXII CCD diffractometer
Absorption correction: multi-scan (*SADABS*; Bruker, 2011 ▶) *T*_min_ = 0.521, *T*_max_ = 0.746
12658 measured reflections
2690 independent reflections
2082 reflections with *I* > 2σ(*I*)
*R*_int_ = 0.057

#### Refinement

*R*[*F* ^2^ > 2σ(*F* ^2^)] = 0.036
*wR*(*F* ^2^) = 0.074
*S* = 1.02
2690 reflections
138 parameters
H-atom parameters constrained
Δρ_max_ = 1.31 e Å^−3^
Δρ_min_ = −0.60 e Å^−3^

### 

Data collection: *APEX2* (Bruker, 2011 ▶); cell refinement: *SAINT* (Bruker, 2011 ▶); data reduction: *SAINT*; program(s) used to solve structure: *SHELXS97* (Sheldrick, 2008 ▶); program(s) used to refine structure: *SHELXL97* (Sheldrick, 2008 ▶); molecular graphics: *CrystalMaker* (CrystalMaker Software, 2009 ▶); software used to prepare material for publication: *enCIFer* (Allen *et al.*, 2004 ▶).

## Supplementary Material

Crystal structure: contains datablock(s) I, global. DOI: 10.1107/S1600536813016371/su2599sup1.cif

Structure factors: contains datablock(s) I. DOI: 10.1107/S1600536813016371/su2599Isup2.hkl

Supplementary material file. DOI: 10.1107/S1600536813016371/su2599Isup3.cml

Additional supplementary materials:  crystallographic information; 3D view; checkCIF report

## Figures and Tables

**Table 1:** Hydrogen-bond geometry (Å, °)

*D*—H⋯*A*	*D*—H	H⋯*A*	*D*⋯*A*	*D*—H⋯*A*
O1—H1⋯O2^i^	0.84	1.80	2.632 (4)	171
O2—H2⋯O1^ii^	0.84	2.01	2.809 (4)	159

Symmetry codes: (i) 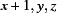; (ii) 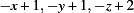.

**Fig. 1.**
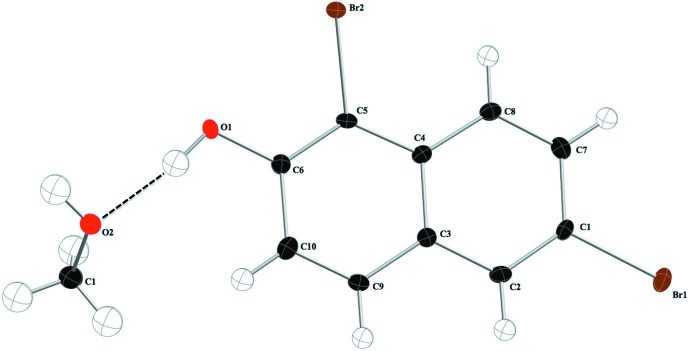
The molecular structure of the title compound, with atom labelling. Displacement ellipsoids are drawn at the 50% probability level. The intermolecular O—H···O hydrogen bond is shown as a dashed line.

**Fig. 2.**
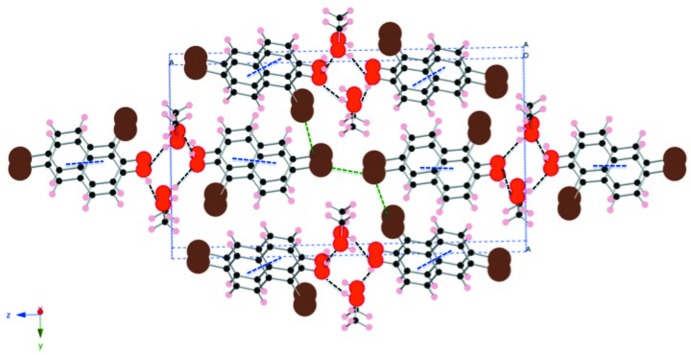
A view along the a axis of the crystal packing of the title compound. The tetramer of Br···Br contacts, the π stacking of the naphthol rings, and the O—H···O hydrogen bonds (Table 1) are show as green, blue and black dashed lines, respectively.

## supplementary crystallographic information

### Comment

Naphthol-containing compounds have gained popularity recently in the pharmaceutical industry as they have potential applications in the synthesis of antipsychotic medications (Costa *et al.*, 2012). The title compound has unique applications as a peroxidase enhancer in peroxidase-catalyzed oxidation reactions (Takeuchi *et al.*, 2000). It is also used to stabilize the two-component system for chemiluminescent assay in immunodiagostics (Kalra & Kumar, 2005).

The molecule of the title compound is planar to within 0.069 (1) Å for all non-H atoms (Fig. 1).

In the crystal, molecules are linked by two pairs of O—H···O hydrogen bonds, involving the methanol molecule, forming dimer-like arrangements (Table 1 and Fig. 2).

The crystal network is further stabilized by π stacking of the naphthol rings with a *Cg*1···*Cg*2^i^ centroid-centroid distance of 3.676 (2) Å [*Cg*1 and *Cg*2 are the centroids of rings C1—C4/C7/C8 and C3—C6/C9/C10, respectively; symmetry code:(i) *x* - 1, *y*, *z*] (see Fig. 2). The crystal structure is also composed of a tetramer of Br···Br contacts, which measure 3.480 (1) Å [Br1···Br1^ii^; symmetry code: (ii) -*x*, -*y* + 1, -*z* + 1] and 3.786 (1) Å [Br2···Br1^iii^; symmetry code: (iii) -*x* + 1/2, *y* + 1/2, -*z* + 3/2]. These contacts are within the normal range of Br···Br interactions, which are typically 3.05 Å to 3.80 Å (Zordan & Brammer, 2006; Schlueter *et al.*, 2012; Desiraju & Parthasarathy, 1989).

### Experimental

Approximately 100 mg of 1,6-dibromo-2-napthol (Sigma-Aldrich) was dissolved in a 2 ml 50% methanol: 50% hexanes solution. On slow evaporation over the course of two weeks colourless plate-like crystals were obtained. The crystals decomposed rapidly when removed from the mother liquor.

### Refinement

The OH and C-bound H atoms were included in calculate positions and treated as riding atoms: O—H = 0.84 Å, C—H = 0.95 and 0.98 Å for CH and CH_3_ H atoms, respectively, with *U*_iso_(H) = 1.5*U*_eq_(*O*,*C*-methyl) and = 1.2*U*_eq_(C) for other H atoms. A residual density peak of 1.31 e/Å^3^ was located near atom C10. Twinning was not found and no disorder could be modeled.

### Crystal data

**Table:**

C_10_H_6_Br_2_O·CH_4_O	*F*(000) = 648
*M**_r_* = 334.01	*D*_x_ = 1.983 Mg m^−^^3^
Monoclinic, *P*2_1_/*n*	Mo *K*α radiation, λ = 0.71073 Å
Hall symbol: P 2yn	Cell parameters from 125 reflections
*a* = 3.9971 (4) Å	θ = 5.5–28.9°
*b* = 12.4705 (12) Å	µ = 7.22 mm^−^^1^
*c* = 22.462 (2) Å	*T* = 100 K
β = 92.442 (1)°	Plate, colourless
*V* = 1118.62 (19) Å^3^	0.26 × 0.11 × 0.01 mm
*Z* = 4	

### Data collection

**Table:**

Bruker APEXII CCD diffractometer	2690 independent reflections
Radiation source: fine-focus sealed tube	2082 reflections with *I* > 2σ(*I*)
Graphite monochromator	*R*_int_ = 0.057
Detector resolution: 8.3333 pixels mm^-1^	θ_max_ = 28.5°, θ_min_ = 1.8°
ω and φ scans	*h* = −5→5
Absorption correction: multi-scan (*SADABS*; Bruker, 2011)	*k* = −16→15
*T*_min_ = 0.521, *T*_max_ = 0.746	*l* = −29→29
12658 measured reflections	

### Refinement

**Table:**

Refinement on *F*^2^	Primary atom site location: structure-invariant direct methods
Least-squares matrix: full	Secondary atom site location: difference Fourier map
*R*[*F*^2^ > 2σ(*F*^2^)] = 0.036	Hydrogen site location: inferred from neighbouring sites
*wR*(*F*^2^) = 0.074	H-atom parameters constrained
*S* = 1.02	*w* = 1/[σ^2^(*F*_o_^2^) + (0.0264*P*)^2^ + 1.7105*P*] where *P* = (*F*_o_^2^ + 2*F*_c_^2^)/3
2690 reflections	(Δ/σ)_max_ = 0.002
138 parameters	Δρ_max_ = 1.31 e Å^−^^3^
0 restraints	Δρ_min_ = −0.60 e Å^−^^3^

### Special details

**Table:**

Geometry. Bond distances, angles *etc*. have been calculated using the rounded fractional coordinates. All su's are estimated from the variances of the (full) variance-covariance matrix. The cell e.s.d.'s are taken into account in the estimation of distances, angles and torsion angles
Refinement. Refinement of *F*^2^ against ALL reflections. The weighted *R*-factor *wR* and goodness of fit *S* are based on *F*^2^, conventional *R*-factors *R* are based on *F*, with *F* set to zero for negative *F*^2^. The threshold expression of *F*^2^ > σ(*F*^2^) is used only for calculating *R*-factors(gt) *etc*. and is not relevant to the choice of reflections for refinement. *R*-factors based on *F*^2^ are statistically about twice as large as those based on *F*, and *R*- factors based on ALL data will be even larger.

### Fractional atomic coordinates and isotropic or equivalent isotropic displacement parameters (Å^2^)

**Table:**

	*x*	*y*	*z*	*U*_iso_*/*U*_eq_	
Br1	0.11971 (11)	0.48530 (3)	0.57493 (2)	0.0238 (1)	
Br2	0.46378 (10)	0.69738 (3)	0.87432 (2)	0.0191 (1)	
O1	0.8338 (7)	0.50830 (19)	0.92461 (11)	0.0218 (9)	
C1	0.2711 (9)	0.5134 (3)	0.65501 (16)	0.0160 (11)	
C2	0.4589 (9)	0.4385 (3)	0.68511 (17)	0.0181 (11)	
C3	0.5579 (9)	0.4567 (3)	0.74594 (17)	0.0154 (11)	
C4	0.4629 (9)	0.5538 (3)	0.77388 (16)	0.0158 (10)	
C5	0.5664 (9)	0.5675 (3)	0.83471 (17)	0.0155 (10)	
C6	0.7448 (9)	0.4917 (3)	0.86645 (17)	0.0176 (11)	
C7	0.1771 (9)	0.6095 (3)	0.68189 (17)	0.0185 (11)	
C8	0.2723 (9)	0.6286 (3)	0.74024 (17)	0.0178 (11)	
C9	0.7470 (9)	0.3789 (3)	0.77869 (17)	0.0175 (11)	
C10	0.8383 (9)	0.3942 (3)	0.83702 (17)	0.0197 (11)	
O2	0.1551 (8)	0.3491 (2)	0.97853 (13)	0.0287 (9)	
C11	−0.0041 (11)	0.2471 (3)	0.98332 (19)	0.0286 (14)	
H1	0.94170	0.45510	0.93800	0.0330*	
H2A	0.52360	0.37460	0.66570	0.0220*	
H7	0.04810	0.66100	0.65980	0.0220*	
H8	0.20790	0.69390	0.75830	0.0210*	
H9	0.81130	0.31480	0.75950	0.0210*	
H10	0.96350	0.34080	0.85830	0.0240*	
H2	0.17580	0.37740	1.01240	0.0430*	
H11A	0.11630	0.20400	1.01380	0.0430*	
H11B	−0.00170	0.20990	0.94490	0.0430*	
H11C	−0.23620	0.25730	0.99460	0.0430*	

### Atomic displacement parameters (Å^2^)

**Table:**

	*U*^11^	*U*^22^	*U*^33^	*U*^12^	*U*^13^	*U*^23^
Br1	0.0299 (2)	0.0256 (2)	0.0156 (2)	−0.0058 (2)	−0.0031 (2)	0.0001 (2)
Br2	0.0230 (2)	0.0152 (2)	0.0191 (2)	0.0037 (2)	−0.0005 (2)	−0.0024 (2)
O1	0.0330 (17)	0.0166 (13)	0.0152 (14)	0.0041 (11)	−0.0052 (12)	0.0022 (11)
C1	0.0176 (19)	0.0182 (18)	0.0122 (18)	−0.0078 (15)	0.0007 (15)	−0.0009 (14)
C2	0.019 (2)	0.0136 (17)	0.022 (2)	−0.0052 (15)	0.0031 (16)	−0.0035 (15)
C3	0.0129 (18)	0.0165 (17)	0.017 (2)	−0.0035 (14)	0.0041 (15)	0.0011 (14)
C4	0.0148 (18)	0.0159 (17)	0.0168 (19)	−0.0043 (14)	0.0028 (15)	−0.0015 (15)
C5	0.0137 (18)	0.0121 (16)	0.021 (2)	0.0005 (14)	0.0032 (15)	−0.0023 (14)
C6	0.0181 (19)	0.0160 (17)	0.0187 (19)	−0.0041 (15)	−0.0001 (15)	0.0011 (15)
C7	0.0165 (19)	0.0158 (18)	0.023 (2)	−0.0040 (15)	0.0002 (16)	0.0050 (15)
C8	0.018 (2)	0.0156 (17)	0.020 (2)	−0.0036 (14)	0.0039 (16)	−0.0027 (15)
C9	0.0171 (19)	0.0135 (17)	0.022 (2)	0.0002 (14)	0.0029 (16)	−0.0008 (15)
C10	0.0159 (19)	0.024 (2)	0.019 (2)	−0.0074 (15)	−0.0001 (16)	−0.0015 (16)
O2	0.0408 (18)	0.0207 (14)	0.0239 (16)	0.0050 (13)	−0.0063 (14)	−0.0016 (12)
C11	0.034 (3)	0.025 (2)	0.027 (2)	0.0026 (18)	0.0030 (19)	0.0009 (18)

### Geometric parameters (Å, º)

**Table:**

Br1—C1	1.906 (4)	C5—C6	1.366 (5)
Br2—C5	1.901 (4)	C6—C10	1.441 (5)
O1—C6	1.355 (5)	C7—C8	1.370 (5)
O1—H1	0.8400	C9—C10	1.359 (5)
O2—C11	1.428 (5)	C2—H2A	0.9500
O2—H2	0.8400	C7—H7	0.9500
C1—C2	1.360 (5)	C8—H8	0.9500
C1—C7	1.400 (5)	C9—H9	0.9500
C2—C3	1.424 (5)	C10—H10	0.9500
C3—C9	1.417 (5)	C11—H11A	0.9800
C3—C4	1.423 (5)	C11—H11B	0.9800
C4—C5	1.421 (5)	C11—H11C	0.9800
C4—C8	1.404 (5)		
			
C6—O1—H1	110.00	C4—C8—C7	121.4 (3)
C11—O2—H2	109.00	C3—C9—C10	121.3 (3)
Br1—C1—C7	119.0 (3)	C6—C10—C9	119.8 (3)
Br1—C1—C2	119.3 (3)	C3—C2—H2A	120.00
C2—C1—C7	121.7 (3)	C1—C2—H2A	120.00
C1—C2—C3	119.5 (3)	C1—C7—H7	120.00
C2—C3—C4	119.4 (3)	C8—C7—H7	120.00
C2—C3—C9	120.6 (3)	C7—C8—H8	119.00
C4—C3—C9	120.0 (3)	C4—C8—H8	119.00
C3—C4—C8	118.5 (3)	C3—C9—H9	119.00
C3—C4—C5	117.0 (3)	C10—C9—H9	119.00
C5—C4—C8	124.5 (3)	C6—C10—H10	120.00
Br2—C5—C6	117.6 (3)	C9—C10—H10	120.00
C4—C5—C6	122.9 (3)	O2—C11—H11A	109.00
Br2—C5—C4	119.5 (3)	O2—C11—H11B	110.00
O1—C6—C10	120.6 (3)	O2—C11—H11C	109.00
C5—C6—C10	119.0 (3)	H11A—C11—H11B	109.00
O1—C6—C5	120.5 (3)	H11A—C11—H11C	109.00
C1—C7—C8	119.5 (3)	H11B—C11—H11C	110.00
			
Br1—C1—C2—C3	−176.7 (3)	C3—C4—C5—C6	−1.0 (5)
C7—C1—C2—C3	1.3 (6)	C8—C4—C5—Br2	−3.1 (5)
Br1—C1—C7—C8	177.2 (3)	C8—C4—C5—C6	178.3 (4)
C2—C1—C7—C8	−0.8 (6)	C3—C4—C8—C7	0.2 (5)
C1—C2—C3—C4	−1.0 (5)	C5—C4—C8—C7	−179.1 (4)
C1—C2—C3—C9	178.6 (3)	Br2—C5—C6—O1	2.1 (5)
C2—C3—C4—C5	179.6 (3)	Br2—C5—C6—C10	−177.5 (3)
C2—C3—C4—C8	0.2 (5)	C4—C5—C6—O1	−179.2 (3)
C9—C3—C4—C5	0.1 (5)	C4—C5—C6—C10	1.2 (6)
C9—C3—C4—C8	−179.3 (3)	O1—C6—C10—C9	−180.0 (3)
C2—C3—C9—C10	−178.8 (4)	C5—C6—C10—C9	−0.3 (5)
C4—C3—C9—C10	0.7 (6)	C1—C7—C8—C4	0.0 (6)
C3—C4—C5—Br2	177.6 (3)	C3—C9—C10—C6	−0.6 (6)

### Hydrogen-bond geometry (Å, º)

**Table:**

*D*—H···*A*	*D*—H	H···*A*	*D*···*A*	*D*—H···*A*
O1—H1···O2^i^	0.84	1.80	2.632 (4)	171
O2—H2···O1^ii^	0.84	2.01	2.809 (4)	159

Symmetry codes: (i) *x*+1, *y*, *z*; (ii) −*x*+1, −*y*+1, −*z*+2.
